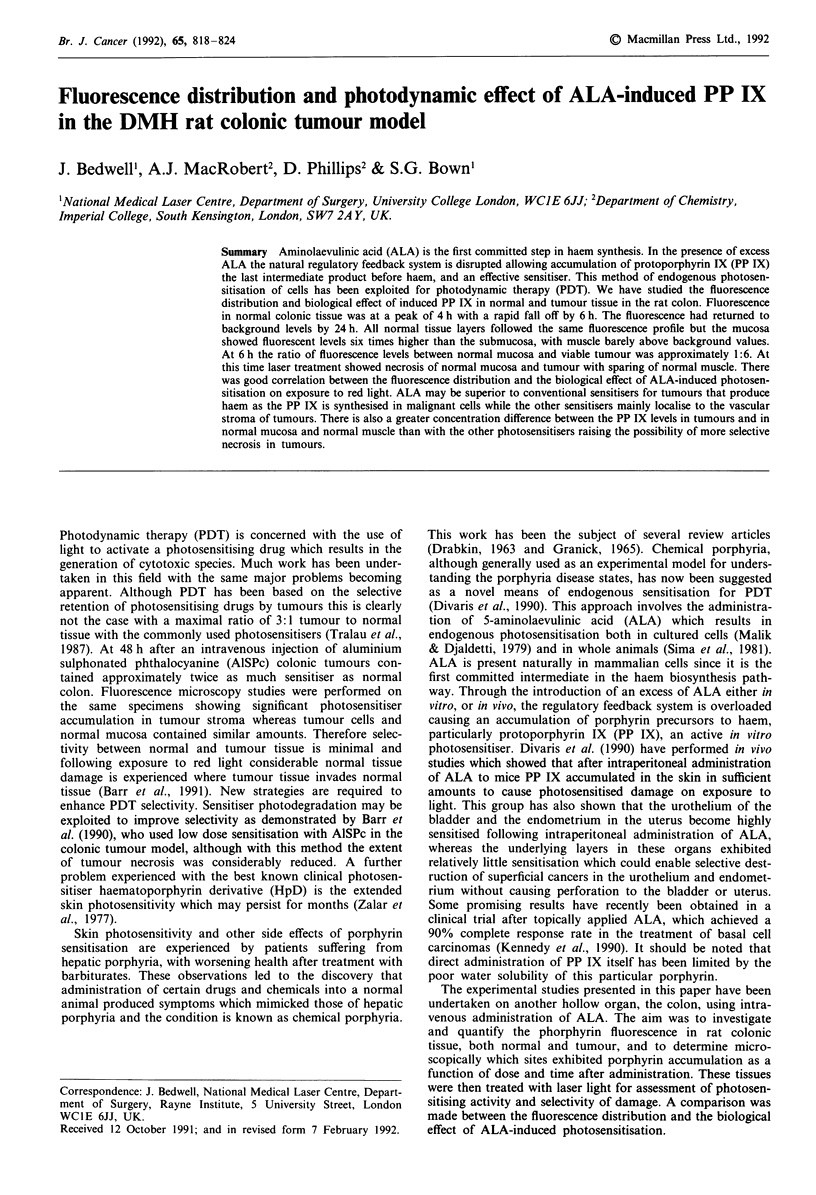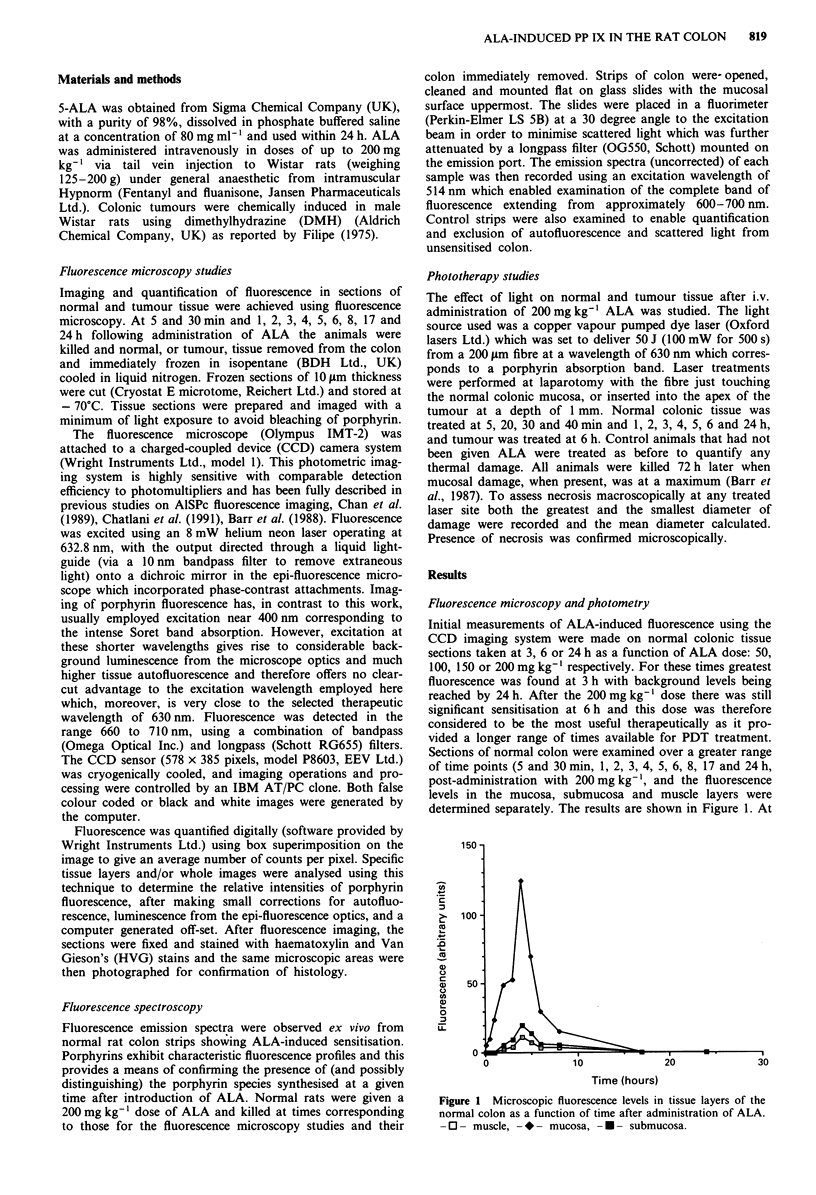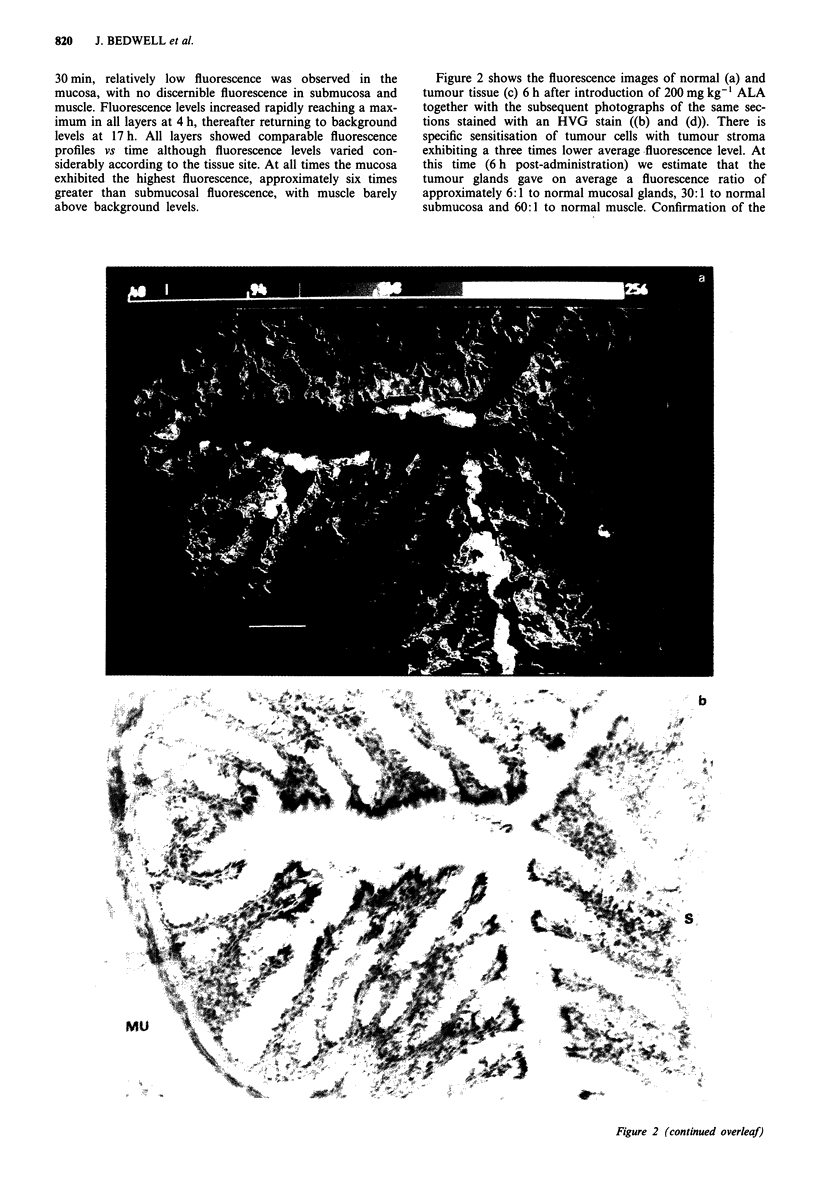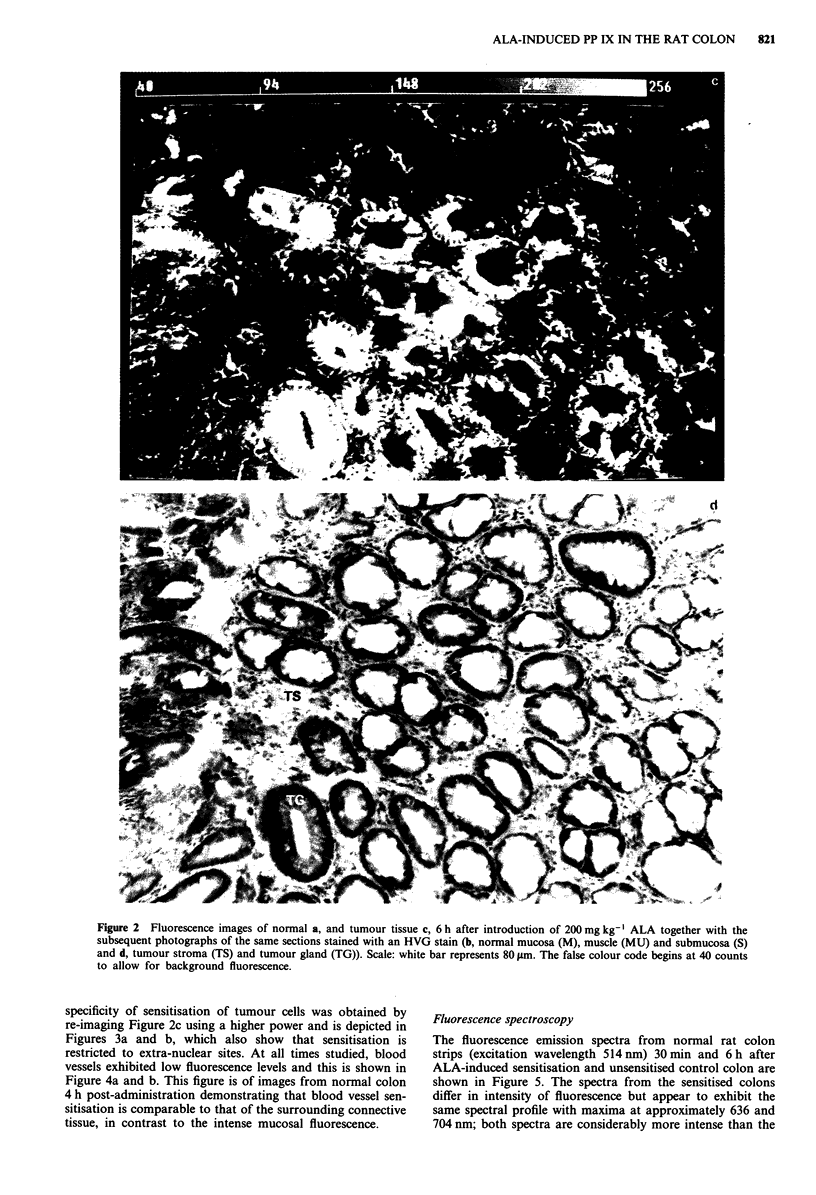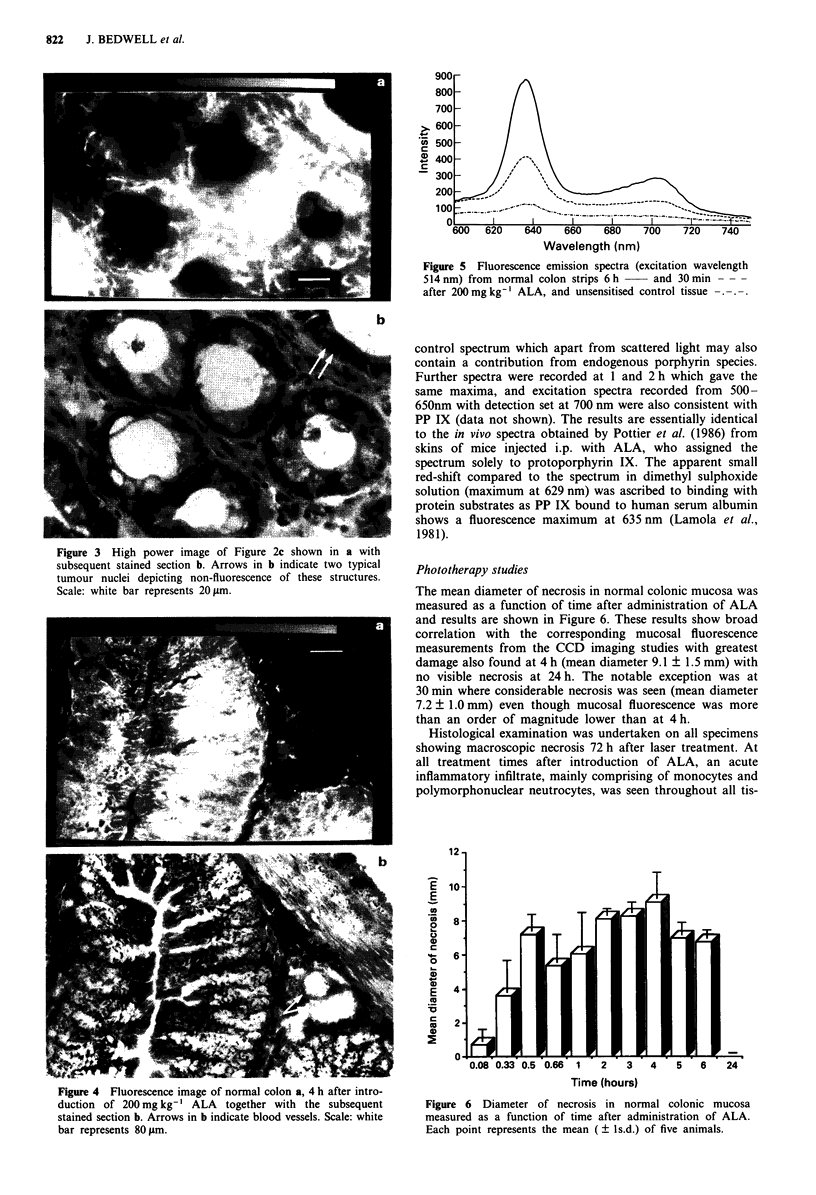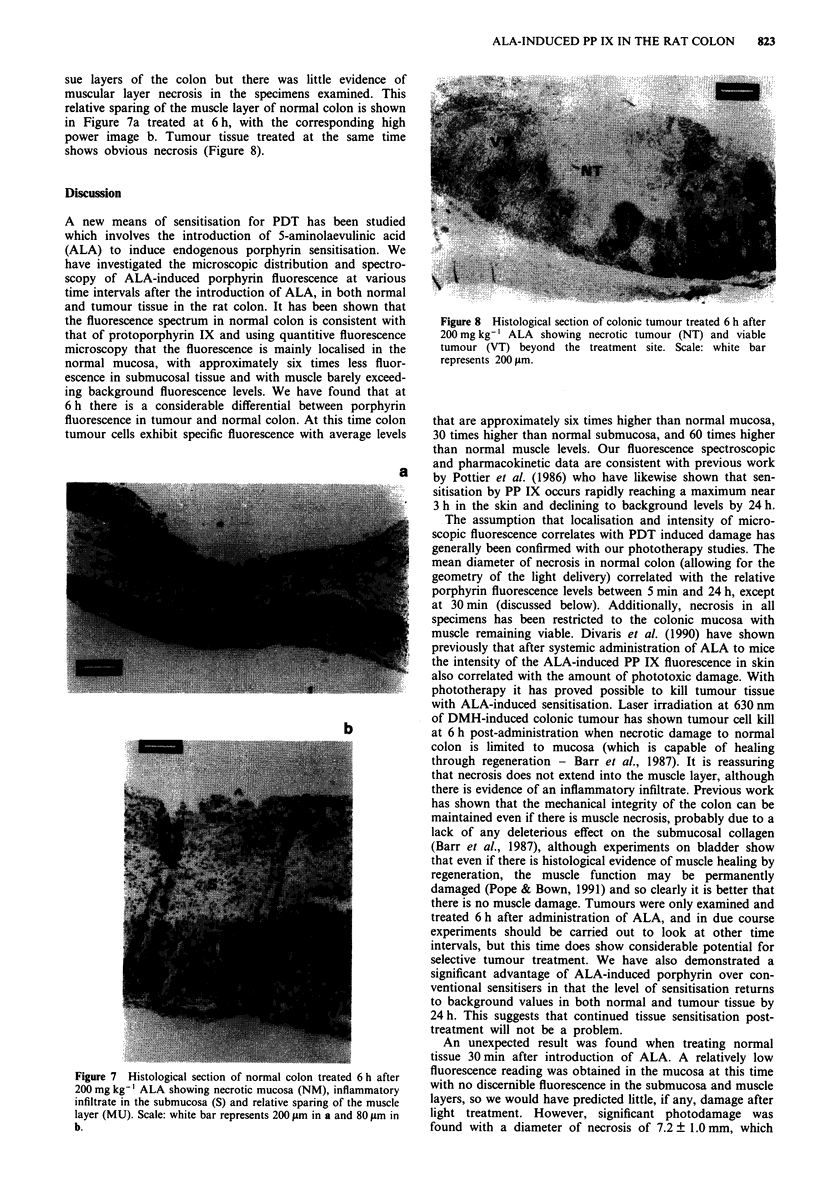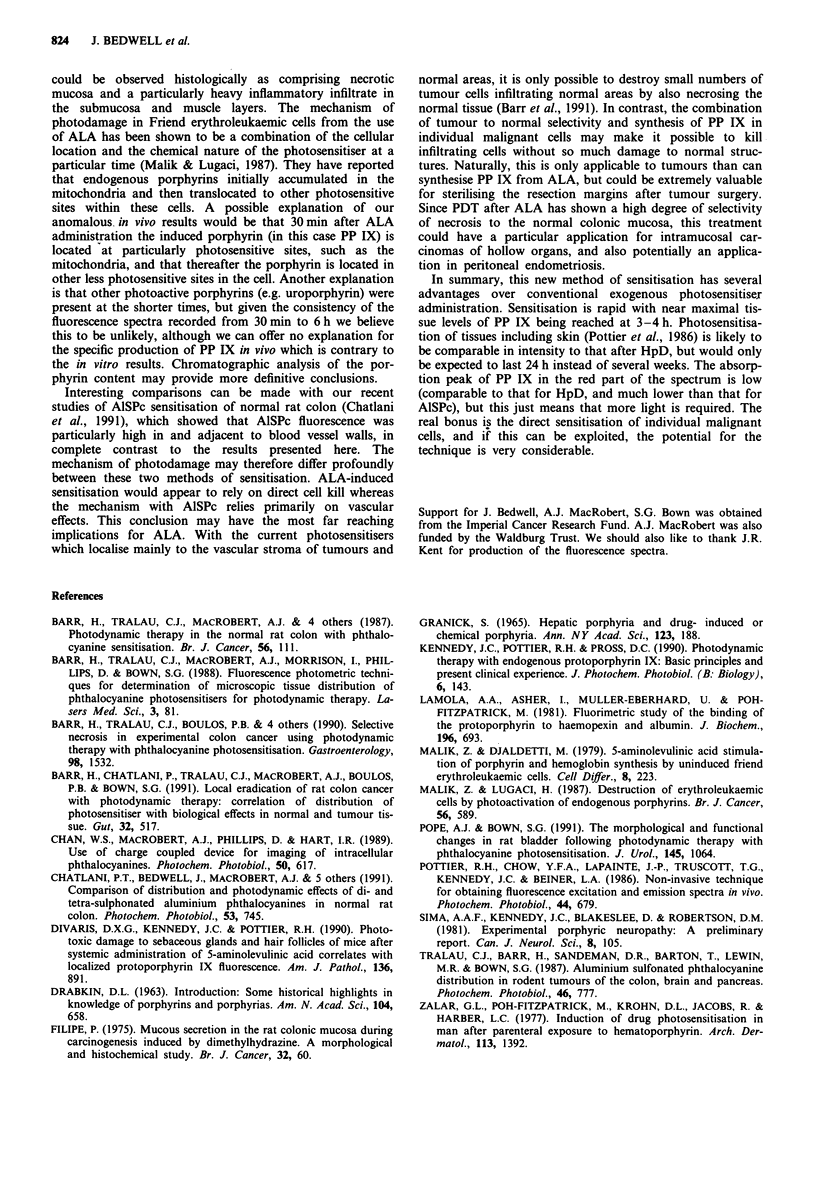# Fluorescence distribution and photodynamic effect of ALA-induced PP IX in the DMH rat colonic tumour model.

**DOI:** 10.1038/bjc.1992.175

**Published:** 1992-06

**Authors:** J. Bedwell, A. J. MacRobert, D. Phillips, S. G. Bown

**Affiliations:** Department of Surgery, University College London, UK.

## Abstract

**Images:**


 © Macmillan Press Ltd., 1992

### Fluorescence distribution and photodynamic effect of ALA-induced PP IX in the DMH rat colonic tumour model

J. Bedwell[1], A.J. MacRobert[2], D. Phillips[2] & S.G. Bown[1]

[1]*National Medical Laser Centre, Department of Surgery, University College London, WC1E 6JJ;* [2]*Department of Chemistry, Imperial College, South Kensington, London, SW7 2AY, UK.*

**Summary** Aminolaevulinic acid (ALA) is the first committed step in haem synthesis. In the presence of excess ALA the natural regulatory feedback system is disrupted allowing accumulation of protoporphyrin IX (PP IX) the last intermediate product before haem, and an effective sensitiser. This method of endogenous photosensitisation of cells has been exploited for photodynamic therapy (PDT). We have studied the fluorescence distribution and biological effect of induced PP IX in normal and tumour tissue in the rat colon. Fluorescence in normal colonic tissue was at a peak of 4 h with a rapid fall off by 6 h. The fluorescence had returned to background levels by 24 h. All normal tissue layers followed the same fluorescence profile but the mucosa showed fluorescent levels six times higher than the submucosa, with muscle barely above background values. At 6 h the ratio of fluorescence levels between normal mucosa and viable tumour was approximately 1:6. At this time laser treatment showed necrosis of normal mucosa and tumour with sparing of normal muscle. There was good correlation between the fluorescence distribution and the biological effect of ALA-induced photosensitisation on exposure to red light. ALA may be superior to conventional sensitisers for tumours that produce haem as the PP IX is synthesised in malignant cells while the other sensitisers mainly localise to the vascular stroma of tumours. There is also a greater concentration difference between the PP IX levels in tumours and in normal mucosa and normal muscle than with the other photosensitisers raising the possibility of more selective necrosis in tumours.

Photodynamic therapy (PDT) is concerned with the use of light to activate a photosensitising drug which results in the generation of cytotoxic species. Much work has been undertaken in this field with the same major problems becoming apparent. Although PDT has been based on the selective retention of photosensitising drugs by tumours this is clearly not the case with a maximal ratio of 3:1 tumour to normal tissue with the commonly used photosensitisers (Tralau *et al.*, 1987). At 48 h after an intravenous injection of aluminium sulphonated phthalocyanine (AlSPc) colonic tumours contained approximately twice as much sensitiser as normal colon. Fluorescence microscopy studies were performed on the same specimens showing significant photosensitiser accumulation in tumour stroma whereas tumour cells and normal mucosa contained similar amounts. Therefore selectivity between normal and tumour tissue is minimal and following exposure to red light considerable normal tissue damage is experienced where tumour tissue invades normal tissue (Barr *et al.*, 1991). New strategies are required to enhance PDT selectivity. Sensitiser photodegradation may be exploited to improve selectivity as demonstrated by Barr *et al.* (1990), who used low dose sensitisation with AlSPc in the colonic tumour model, although with this method the extent of tumour necrosis was considerably reduced. A further problem experienced with the best known clinical photosensitiser haematoporphyrin derivative (HpD) is the extended skin photosensitivity which may persist for months (Zalar *et al.*, 1977).

Skin photosensitivity and other side effects of porphyrin sensitisation are experienced by patients suffering from hepatic porphyria, with worsening health after treatment with barbiturates. These observations led to the discovery that administration of certain drugs and chemicals into a normal animal produced symptoms which mimicked those of hepatic porphyria and the condition is known as chemical porphyria. This work has been the subject of several review articles (Drabkin, 1963 and Granick, 1965). Chemical porphyria, although generally used as an experimental model for understanding the porphyria disease states, has now been suggested as a novel means of endogenous sensitisation for PDT (Divaris *et al.*, 1990). This approach involves the administration of 5-aminolaevulinic acid (ALA) which results in endogenous photosensitisation both in cultured cells (Malik & Djaldetti, 1979) and in whole animals (Sima *et al.*, 1981). ALA is present naturally in mammalian cells since it is the first committed intermediate in the haem biosynthesis pathway. Through the introduction of an excess of ALA either *in vitro*, or *in vivo*, the regulatory feedback system is overloaded causing an accumulation of porphyrin precursors to haem, particularly protoporphyrin IX (PP IX), an active *in vitro* photosensitiser. Divaris *et al.* (1990) have performed *in vivo* studies which showed that after intraperitoneal administration of ALA to mice PP IX accumulated in the skin in sufficient amounts to cause photosensitised damage on exposure to light. This group has also shown that the urothelium of the bladder and the endometrium in the uterus become highly sensitised following intraperitoneal administration of ALA, whereas the underlying layers in these organs exhibited relatively little sensitisation which could enable selective destruction of superficial cancers in the urothelium and endometrium without causing perforation to the bladder or uterus. Some promising results have recently been obtained in a clinical trial after topically applied ALA, which achieved a 90% complete response rate in the treatment of basal cell carcinomas (Kennedy *et al.*, 1990). It should be noted that direct administration of PP IX itself has been limited by the poor water solubility of this particular porphyrin.

The experimental studies presented in this paper have been undertaken on another hollow organ, the colon, using intravenous administration of ALA. The aim was to investigate and quantify the phorphyrin fluorescence in rat colonic tissue, both normal and tumour, and to determine microscopically which sites exhibited porphyrin accumulation as a function of dose and time after administration. These tissues were then treated with laser light for assessment of photosensitising activity and selectivity of damage. A comparison was made between the fluorescence distribution and the biological effect of ALA-induced photosensitisation.

Correspondence: J. Bedwell, National Medical Laser Centre, Department of Surgery, Rayne Institute, 5 University Street, London WC1E 6JJ, UK.
Received 12 October 1991; and in revised form 7 February 1992.

### Materials and methods

5-ALA was obtained from Sigma Chemical Company (UK), with a purity of 98%, dissolved in phosphate buffered saline at a concentration of 80 mg $ml^{-1}$ and used within 24 h. ALA was administered intravenously in doses of up to 200 mg $kg^{-1}$ via tail vein injection to Wistar rats (weighing 125–200 g) under general anaesthetic from intramuscular Hypnorm (Fentanyl and fluanisone, Jansen Pharmaceuticals Ltd.). Colonic tumours were chemically induced in male Wistar rats using dimethylhydrazine (DMH) (Aldrich Chemical Company, UK) as reported by Filipe (1975).

### *Fluorescence microscopy studies*

Imaging and quantification of fluorescence in sections of normal and tumour tissue were achieved using fluorescence microscopy. At 5 and 30 min and 1, 2, 3, 4, 5, 6, 8, 17 and 24 h following administration of ALA the animals were killed and normal, or tumour, tissue removed from the colon and immediately frozen in isopentane (BDH Ltd., UK) cooled in liquid nitrogen. Frozen sections of 10 µm thickness were cut (Cryostat E microtome, Reichert Ltd.) and stored at – 70°C. Tissue sections were prepared and imaged with a minimum of light exposure to avoid bleaching of porphyrin.

The fluorescence microscope (Olympus IMT-2) was attached to a charged-coupled device (CCD) camera system (Wright Instruments Ltd., model 1). This photometric imaging system is highly sensitive with comparable detection efficiency to photomultipliers and has been fully described in previous studies on AlSPc fluorescence imaging, Chan *et al.* (1989), Chatlani *et al.* (1991), Barr *et al.* (1988). Fluorescence was excited using an 8 mW helium neon laser operating at 632.8 nm, with the output directed through a liquid light-guide (via a 10 nm bandpass filter to remove extraneous light) onto a dichroic mirror in the epi-fluorescence microscope which incorporated phase-contrast attachments. Imaging of porphyrin fluorescence has, in contrast to this work, usually employed excitation near 400 nm corresponding to the intense Soret band absorption. However, excitation at these shorter wavelengths gives rise to considerable background luminescence from the microscope optics and much higher tissue autofluorescence and therefore offers no clear-cut advantage to the excitation wavelength employed here which, moreover, is very close to the selected therapeutic wavelength of 630 nm. Fluorescence was detected in the range 660 to 710 nm, using a combination of bandpass (Omega Optical Inc.) and longpass (Schott RG655) filters. The CCD sensor (578 × 385 pixels, model P8603, EEV Ltd.) was cryogenically cooled, and imaging operations and processing were controlled by an IBM AT/PC clone. Both false colour coded or black and white images were generated by the computer.

Fluorescence was quantified digitally (software provided by Wright Instruments Ltd.) using box superimposition on the image to give an average number of counts per pixel. Specific tissue layers and/or whole images were analysed using this technique to determine the relative intensities of porphyrin fluorescence, after making small corrections for autofluorescence, luminescence from the epi-fluorescence optics, and a computer generated off-set. After fluorescence imaging, the sections were fixed and stained with haematoxylin and Van Gieson's (HVG) stains and the same microscopic areas were then photographed for confirmation of histology.

### *Fluorescence spectroscopy*

Fluorescence emission spectra were observed *ex vivo* from normal rat colon strips showing ALA-induced sensitisation. Porphyrins exhibit characteristic fluorescence profiles and this provides a means of confirming the presence of (and possibly distinguishing) the porphyrin species synthesised at a given time after introduction of ALA. Normal rats were given a 200 mg $kg^{-1}$ dose of ALA and killed at times corresponding to those for the fluorescence microscopy studies and their colon immediately removed. Strips of colon were opened, cleaned and mounted flat on glass slides with the mucosal surface uppermost. The slides were placed in a fluorimeter (Perkin-Elmer LS 5B) at a 30 degree angle to the excitation beam in order to minimise scattered light which was further attenuated by a longpass filter (OG550, Schott) mounted on the emission port. The emission spectra (uncorrected) of each sample was then recorded using an excitation wavelength of 514 nm which enabled examination of the complete band of fluorescence extending from approximately 600–700 nm. Control strips were also examined to enable quantification and exclusion of autofluorescence and scattered light from unsensitised colon.

### *Phototherapy studies*

The effect of light on normal and tumour tissue after i.v. administration of 200 mg $kg^{-1}$ ALA was studied. The light source used was a copper vapour pumped dye laser (Oxford lasers Ltd.) which was set to deliver 50 J (100 mW for 500 s) from a 200 µm fibre at a wavelength of 630 nm which corresponds to a porphyrin absorption band. Laser treatments were performed at laparotomy with the fibre just touching the normal colonic mucosa, or inserted into the apex of the tumour at a depth of 1 mm. Normal colonic tissue was treated at 5, 20, 30 and 40 min and 1, 2, 3, 4, 5, 6 and 24 h, and tumour was treated at 6 h. Control animals that had not been given ALA were treated as before to quantify any thermal damage. All animals were killed 72 h later when mucosal damage, when present, was at a maximum (Barr *et al.*, 1987). To assess necrosis macroscopically at any treated laser site both the greatest and the smallest diameter of damage were recorded and the mean diameter calculated. Presence of necrosis was confirmed microscopically.

### Results

### *Fluorescence microscopy and photometry*

Initial measurements of ALA-induced fluorescence using the CCD imaging system were made on normal colonic tissue sections taken at 3, 6 or 24 h as a function of ALA dose: 50, 100, 150 or 200 mg $kg^{-1}$ respectively. For these times greatest fluorescence was found at 3 h with background levels being reached by 24 h. After the 200 mg $kg^{-1}$ dose there was still significant sensitisation at 6 h and this dose was therefore considered to be the most useful therapeutically as it provided a longer range of times available for PDT treatment. Sections of normal colon were examined over a greater range of time points (5 and 30 min, 1, 2, 3, 4, 5, 6, 8, 17 and 24 h, post-administration with 200 mg $kg^{-1}$, and the fluorescence levels in the mucosa, submucosa and muscle layers were determined separately. The results are shown in Figure 1. At

**Figure 1** Microscopic fluorescence levels in tissue layers of the normal colon as a function of time after administration of ALA. –□– muscle, –◆– mucosa, –■– submucosa.

30 min, relatively low fluorescence was observed in the mucosa, with no discernible fluorescence in submucosa and muscle. Fluorescence levels increased rapidly reaching a maximum in all layers at 4 h, thereafter returning to background levels at 17 h. All layers showed comparable fluorescence profiles *vs* time although fluorescence levels varied considerably according to the tissue site. At all times the mucosa exhibited the highest fluorescence, approximately six times greater than submucosal fluorescence, with muscle barely above background levels.

Figure 2 shows the fluorescence images of normal (a) and tumour tissue (c) 6 h after introduction of 200 mg kg$^{-1}$ ALA together with the subsequent photographs of the same sections stained with an HVG stain ((b) and (d)). There is specific sensitisation of tumour cells with tumour stroma exhibiting a three times lower average fluorescence level. At this time (6 h post-administration) we estimate that the tumour glands gave on average a fluorescence ratio of approximately 6:1 to normal mucosal glands, 30:1 to normal submucosa and 60:1 to normal muscle. Confirmation of the

*Figure 2 (continued overleaf)*

**Figure 2** Fluorescence images of normal **a**, and tumour tissue **c**, 6 h after introduction of 200 mg $kg^{-1}$ ALA together with the subsequent photographs of the same sections stained with an HVG stain (**b**, normal mucosa (M), muscle (MU) and submucosa (S) and **d**, tumour stroma (TS) and tumour gland (TG)). Scale: white bar represents 80 $\mu$m. The false colour code begins at 40 counts to allow for background fluorescence.

specificity of sensitisation of tumour cells was obtained by re-imaging Figure 2c using a higher power and is depicted in Figures 3a and b, which also show that sensitisation is restricted to extra-nuclear sites. At all times studied, blood vessels exhibited low fluorescence levels and this is shown in Figure 4a and b. This figure is of images from normal colon 4 h post-administration demonstrating that blood vessel sensitisation is comparable to that of the surrounding connective tissue, in contrast to the intense mucosal fluorescence.

*Fluorescence spectroscopy*

The fluorescence emission spectra from normal rat colon strips (excitation wavelength 514 nm) 30 min and 6 h after ALA-induced sensitisation and unsensitised control colon are shown in Figure 5. The spectra from the sensitised colons differ in intensity of fluorescence but appear to exhibit the same spectral profile with maxima at approximately 636 and 704 nm; both spectra are considerably more intense than the

**Figure 3** High power image of Figure 2c shown in **a** with subsequent stained section **b**. Arrows in **b** indicate two typical tumour nuclei depicting non-fluorescence of these structures. Scale: white bar represents 20 μm.

**Figure 4** Fluorescence image of normal colon **a**, 4 h after introduction of 200 mg kg$^{-1}$ ALA together with the subsequent stained section **b**. Arrows in **b** indicate blood vessels. Scale: white bar represents 80 μm.

**Figure 5** Fluorescence emission spectra (excitation wavelength 514 nm) from normal colon strips 6 h —— and 30 min – – – after 200 mg kg$^{-1}$ ALA, and unsensitised control tissue –.–.–.

control spectrum which apart from scattered light may also contain a contribution from endogenous porphyrin species. Further spectra were recorded at 1 and 2 h which gave the same maxima, and excitation spectra recorded from 500–650nm with detection set at 700 nm were also consistent with PP IX (data not shown). The results are essentially identical to the *in vivo* spectra obtained by Pottier *et al.* (1986) from skins of mice injected i.p. with ALA, who assigned the spectrum solely to protoporphyrin IX. The apparent small red-shift compared to the spectrum in dimethyl sulphoxide solution (maximum at 629 nm) was ascribed to binding with protein substrates as PP IX bound to human serum albumin shows a fluorescence maximum at 635 nm (Lamola *et al.*, 1981).

### *Phototherapy studies*

The mean diameter of necrosis in normal colonic mucosa was measured as a function of time after administration of ALA and results are shown in Figure 6. These results show broad correlation with the corresponding mucosal fluorescence measurements from the CCD imaging studies with greatest damage also found at 4 h (mean diameter 9.1 ± 1.5 mm) with no visible necrosis at 24 h. The notable exception was at 30 min where considerable necrosis was seen (mean diameter 7.2 ± 1.0 mm) even though mucosal fluorescence was more than an order of magnitude lower than at 4 h.

Histological examination was undertaken on all specimens showing macroscopic necrosis 72 h after laser treatment. At all treatment times after introduction of ALA, an acute inflammatory infiltrate, mainly comprising of monocytes and polymorphonuclear neutrocytes, was seen throughout all tis-

**Figure 6** Diameter of necrosis in normal colonic mucosa measured as a function of time after administration of ALA. Each point represents the mean (± 1s.d.) of five animals.

sue layers of the colon but there was little evidence of muscular layer necrosis in the specimens examined. This relative sparing of the muscle layer of normal colon is shown in Figure 7a treated at 6 h, with the corresponding high power image b. Tumour tissue treated at the same time shows obvious necrosis (Figure 8).

### Discussion

A new means of sensitisation for PDT has been studied which involves the introduction of 5-aminolaevulinic acid (ALA) to induce endogenous porphyrin sensitisation. We have investigated the microscopic distribution and spectroscopy of ALA-induced porphyrin fluorescence at various time intervals after the introduction of ALA, in both normal and tumour tissue in the rat colon. It has been shown that the fluorescence spectrum in normal colon is consistent with that of protoporphyrin IX and using quantitive fluorescence microscopy that the fluorescence is mainly localised in the normal mucosa, with approximately six times less fluorescence in submucosal tissue and with muscle barely exceeding background fluorescence levels. We have found that at 6 h there is a considerable differential between porphyrin fluorescence in tumour and normal colon. At this time colon tumour cells exhibit specific fluorescence with average levels

**Figure 7** Histological section of normal colon treated 6 h after 200 mg $kg^{-1}$ ALA showing necrotic mucosa (NM), inflammatory infiltrate in the submucosa (S) and relative sparing of the muscle layer (MU). Scale: white bar represents 200 μm in **a** and 80 μm in **b**.

**Figure 8** Histological section of colonic tumour treated 6 h after 200 mg $kg^{-1}$ ALA showing necrotic tumour (NT) and viable tumour (VT) beyond the treatment site. Scale: white bar represents 200 μm.

that are approximately six times higher than normal mucosa, 30 times higher than normal submucosa, and 60 times higher than normal muscle levels. Our fluorescence spectroscopic and pharmacokinetic data are consistent with previous work by Pottier *et al.* (1986) who have likewise shown that sensitisation by PP IX occurs rapidly reaching a maximum near 3 h in the skin and declining to background levels by 24 h.

The assumption that localisation and intensity of microscopic fluorescence correlates with PDT induced damage has generally been confirmed with our phototherapy studies. The mean diameter of necrosis in normal colon (allowing for the geometry of the light delivery) correlated with the relative porphyrin fluorescence levels between 5 min and 24 h, except at 30 min (discussed below). Additionally, necrosis in all specimens has been restricted to the colonic mucosa with muscle remaining viable. Divaris *et al.* (1990) have shown previously that after systemic administration of ALA to mice the intensity of the ALA-induced PP IX fluorescence in skin also correlated with the amount of phototoxic damage. With phototherapy it has proved possible to kill tumour tissue with ALA-induced sensitisation. Laser irradiation at 630 nm of DMH-induced colonic tumour has shown tumour cell kill at 6 h post-administration when necrotic damage to normal colon is limited to mucosa (which is capable of healing through regeneration – Barr *et al.*, 1987). It is reassuring that necrosis does not extend into the muscle layer, although there is evidence of an inflammatory infiltrate. Previous work has shown that the mechanical integrity of the colon can be maintained even if there is muscle necrosis, probably due to a lack of any deleterious effect on the submucosal collagen (Barr *et al.*, 1987), although experiments on bladder show that even if there is histological evidence of muscle healing by regeneration, the muscle function may be permanently damaged (Pope & Bown, 1991) and so clearly it is better that there is no muscle damage. Tumours were only examined and treated 6 h after administration of ALA, and in due course experiments should be carried out to look at other time intervals, but this time does show considerable potential for selective tumour treatment. We have also demonstrated a significant advantage of ALA-induced porphyrin over conventional sensitisers in that the level of sensitisation returns to background values in both normal and tumour tissue by 24 h. This suggests that continued tissue sensitisation post-treatment will not be a problem.

An unexpected result was found when treating normal tissue 30 min after introduction of ALA. A relatively low fluorescence reading was obtained in the mucosa at this time with no discernible fluorescence in the submucosa and muscle layers, so we would have predicted little, if any, damage after light treatment. However, significant photodamage was found with a diameter of necrosis of 7.2 ± 1.0 mm, which could be observed histologically as comprising necrotic mucosa and a particularly heavy inflammatory infiltrate in the submucosa and muscle layers. The mechanism of photodamage in Friend erythroleukaemic cells from the use of ALA has been shown to be a combination of the cellular location and the chemical nature of the photosensitiser at a particular time (Malik & Lugaci, 1987). They have reported that endogenous porphyrins initially accumulated in the mitochondria and then translocated to other photosensitive sites within these cells. A possible explanation of our anomalous *in vivo* results would be that 30 min after ALA administration the induced porphyrin (in this case PP IX) is located at particularly photosensitive sites, such as the mitochondria, and that thereafter the porphyrin is located in other less photosensitive sites in the cell. Another explanation is that other photoactive porphyrins (e.g. uroporphyrin) were present at the shorter times, but given the consistency of the fluorescence spectra recorded from 30 min to 6 h we believe this to be unlikely, although we can offer no explanation for the specific production of PP IX *in vivo* which is contrary to the *in vitro* results. Chromatographic analysis of the porphyrin content may provide more definitive conclusions.

Interesting comparisons can be made with our recent studies of AlSPc sensitisation of normal rat colon (Chatlani *et al.*, 1991), which showed that AlSPc fluorescence was particularly high in and adjacent to blood vessel walls, in complete contrast to the results presented here. The mechanism of photodamage may therefore differ profoundly between these two methods of sensitisation. ALA-induced sensitisation would appear to rely on direct cell kill whereas the mechanism with AlSPc relies primarily on vascular effects. This conclusion may have the most far reaching implications for ALA. With the current photosensitisers which localise mainly to the vascular stroma of tumours and normal areas, it is only possible to destroy small numbers of tumour cells infiltrating normal areas by also necrosing the normal tissue (Barr *et al.*, 1991). In contrast, the combination of tumour to normal selectivity and synthesis of PP IX in individual malignant cells may make it possible to kill infiltrating cells without so much damage to normal structures. Naturally, this is only applicable to tumours than can synthesise PP IX from ALA, but could be extremely valuable for sterilising the resection margins after tumour surgery. Since PDT after ALA has shown a high degree of selectivity of necrosis to the normal colonic mucosa, this treatment could have a particular application for intramucosal carcinomas of hollow organs, and also potentially an application in peritoneal endometriosis.

In summary, this new method of sensitisation has several advantages over conventional exogenous photosensitiser administration. Sensitisation is rapid with near maximal tissue levels of PP IX being reached at 3–4 h. Photosensitisation of tissues including skin (Pottier *et al.*, 1986) is likely to be comparable in intensity to that after HpD, but would only be expected to last 24 h instead of several weeks. The absorption peak of PP IX in the red part of the spectrum is low (comparable to that for HpD, and much lower than that for AlSPc), but this just means that more light is required. The real bonus is the direct sensitisation of individual malignant cells, and if this can be exploited, the potential for the technique is very considerable.

Support for J. Bedwell, A.J. MacRobert, S.G. Bown was obtained from the Imperial Cancer Research Fund. A.J. MacRobert was also funded by the Waldburg Trust. We should also like to thank J.R. Kent for production of the fluorescence spectra.

### References

BARR, H., TRALAU, C.J., MACROBERT, A.J. & 4 others (1987). Photodynamic therapy in the normal rat colon with phthalocyanine sensitisation. *Br. J. Cancer*, **56**, 111.

BARR, H., TRALAU, C.J., MACROBERT, A.J., MORRISON, I., PHILLIPS, D. & BOWN, S.G. (1988). Fluorescence photometric techniques for determination of microscopic tissue distribution of phthalocyanine photosensitisers for photodynamic therapy. *Lasers Med. Sci.*, **3**, 81.

BARR, H., TRALAU, C.J., BOULOS, P.B. & 4 others (1990). Selective necrosis in experimental colon cancer using photodynamic therapy with phthalocyanine photosensitisation. *Gastroenterology*, **98**, 1532.

BARR, H., CHATLANI, P., TRALAU, C.J., MACROBERT, A.J., BOULOS, P.B. & BOWN, S.G. (1991). Local eradication of rat colon cancer with photodynamic therapy: correlation of distribution of photosensitiser with biological effects in normal and tumour tissue. *Gut*, **32**, 517.

CHAN, W.S., MACROBERT, A.J., PHILLIPS, D. & HART, I.R. (1989). Use of charge coupled device for imaging of intracellular phthalocyanines. *Photochem. Photobiol.*, **50**, 617.

CHATLANI, P.T., BEDWELL, J., MACROBERT, A.J. & 5 others (1991). Comparison of distribution and photodynamic effects of di- and tetra-sulphonated aluminium phthalocyanines in normal rat colon. *Photochem. Photobiol.*, **53**, 745.

DIVARIS, D.X.G., KENNEDY, J.C. & POTTIER, R.H. (1990). Phototoxic damage to sebaceous glands and hair follicles of mice after systemic administration of 5-aminolevulinic acid correlates with localized protoporphyrin IX fluorescence. *Am. J. Pathol.*, **136**, 891.

DRABKIN, D.L. (1963). Introduction: Some historical highlights in knowledge of porphyrins and porphyrias. *Am. N. Acad. Sci.*, **104**, 658.

FILIPE, P. (1975). Mucous secretion in the rat colonic mucosa during carcinogenesis induced by dimethylhydrazine. A morphological and histochemical study. *Br. J. Cancer*, **32**, 60.

GRANICK, S. (1965). Hepatic porphyria and drug- induced or chemical porphyria. *Ann. NY Acad. Sci.*, **123**, 188.

KENNEDY, J.C., POTTIER, R.H. & PROSS, D.C. (1990). Photodynamic therapy with endogenous protoporphyrin IX: Basic principles and present clinical experience. *J. Photochem. Photobiol. (B: Biology)*, **6**, 143.

LAMOLA, A.A., ASHER, I., MULLER-EBERHARD, U. & POH-FITZPATRICK, M. (1981). Fluorimetric study of the binding of the protoporphyrin to haemopexin and albumin. *J. Biochem.*, **196**, 693.

MALIK, Z. & DJALDETTI, M. (1979). 5-aminolevulinic acid stimulation of porphyrin and hemoglobin synthesis by uninduced friend erythroleukaemic cells. *Cell Differ.*, **8**, 223.

MALIK, Z. & LUGACI, H. (1987). Destruction of erythroleukaemic cells by photoactivation of endogenous porphyrins. *Br. J. Cancer*, **56**, 589.

POPE, A.J. & BOWN, S.G. (1991). The morphological and functional changes in rat bladder following photodynamic therapy with phthalocyanine photosensitisation. *J. Urol.*, **145**, 1064.

POTTIER, R.H., CHOW, Y.F.A., LAPAINTE, J.-P., TRUSCOTT, T.G., KENNEDY, J.C. & BEINER, L.A. (1986). Non-invasive technique for obtaining fluorescence excitation and emission spectra *in vivo*. *Photochem. Photobiol.*, **44**, 679.

SIMA, A.A.F., KENNEDY, J.C., BLAKESLEE, D. & ROBERTSON, D.M. (1981). Experimental porphyric neuropathy: A preliminary report. *Can. J. Neurol. Sci.*, **8**, 105.

TRALAU, C.J., BARR, H., SANDEMAN, D.R., BARTON, T., LEWIN, M.R. & BOWN, S.G. (1987). Aluminium sulfonated phthalocyanine distribution in rodent tumours of the colon, brain and pancreas. *Photochem. Photobiol.*, **46**, 777.

ZALAR, G.L., POH-FITZPATRICK, M., KROHN, D.L., JACOBS, R. & HARBER, L.C. (1977). Induction of drug photosensitisation in man after parenteral exposure to hematoporphyrin. *Arch. Dermatol.*, **113**, 1392.